# Analytical Methodologies to Detect N‑Nitrosamine Impurities in Active Pharmaceutical Ingredients, Drug Products and Other Matrices

**DOI:** 10.1021/acs.chemrestox.4c00234

**Published:** 2024-08-19

**Authors:** Krishna Moorthy Manchuri, Mahammad Ali Shaik, Venkata Subba Reddy Gopireddy, Naziya Sultana, Sreenivasarao Gogineni

**Affiliations:** † Department of Chemistry, 470792 Jawaharlal Nehru Technological University Anantapur, Ananthapuramu, Andhra Pradesh 515002, India; ‡ Analytical Research and Development, 28574 IPDO, Dr. Reddy’s Laboratories Limited, Hyderabad 500090, India; § Department of Chemistry, 28629 Acharya Nagarjuna University, Nagarjuna Nagar, Guntur, Andhra Pradesh 522510, India

## Abstract

Since 2018, N-nitrosamine impurities have become a widespread concern in the global regulatory landscape of pharmaceutical products. This concern arises due to their potential for contamination, toxicity, carcinogenicity, and mutagenicity and their presence in many active pharmaceutical ingredients, drug products, and other matrices. N-Nitrosamine impurities in humans can lead to severe chemical toxicity effects. These include carcinogenic effects, metabolic disruptions, reproductive harm, liver diseases, obesity, DNA damage, cell death, chromosomal alterations, birth defects, and pregnancy loss. They are particularly known to cause cancer (tumors) in various organs and tissues such as the liver, lungs, nasal cavity, esophagus, pancreas, stomach, urinary bladder, colon, kidneys, and central nervous system. Additionally, N-nitrosamine impurities may contribute to the development of Alzheimer’s and Parkinson’s diseases and type-2 diabetes. Therefore, it is very important to control or avoid them by enhancing effective analytical methodologies using cutting-edge analytical techniques such as LC-MS, GC-MS, CE-MS, SFC, etc. Moreover, these analytical methods need to be sensitive and selective with suitable precision and accuracy, so that the actual amounts of N-nitrosamine impurities can be detected and quantified appropriately in drugs. Regulatory agencies such as the US FDA, EMA, ICH, WHO, etc. need to focus more on the hazards of N-nitrosamine impurities by providing guidance and regular updates to drug manufacturers and applicants. Similarly, drug manufacturers should be more vigilant to avoid nitrosating agents and secondary amines during the manufacturing processes. Numerous review articles have been published recently by various researchers, focusing on N-nitrosamine impurities found in previously notified products, including sartans, metformin, and ranitidine. These impurities have also been detected in a wide range of other products. Consequently, this review aims to concentrate on products recently reported to contain N-nitrosamine impurities. These products include rifampicin, champix, famotidine, nizatidine, atorvastatin, bumetanide, itraconazole, diovan, enalapril, propranolol, lisinopril, duloxetine, rivaroxaban, pioglitazones, glifizones, cilostazol, and sunitinib.

## Graphical abstract

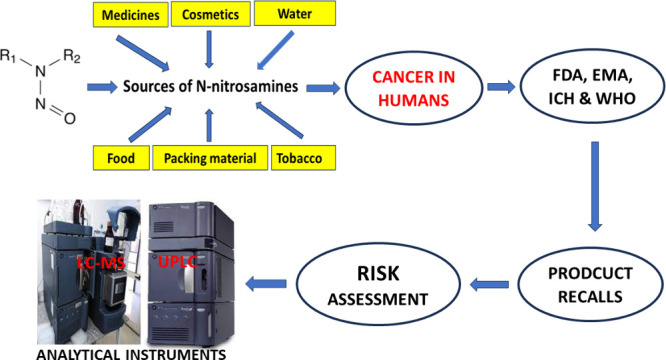

## 1 What Are N-Nitrosamine Impurities

N-Nitrosamine impurities are organic compounds that refer to any molecule containing the nitroso (N—N=O) functional group. In organic chemistry, “nitroso” refers to a functional group in which the nitric oxide (N—N=O) group is attached to an organic moiety. Essentially, nitroso groups can be categorized as C-nitroso compounds (e.g., nitroso alkanes; R—N=O), S-nitroso compounds (nitroso thiols; RS—N=O), N-nitroso compounds (e.g., nitrosamines, RN­(—R′)—N=O), and O-nitroso compounds (alkyl nitrites; RO—N=O). N-Nitrosamines have the general structure shown in Figure 1.^1^ N-Nitroso- impurities are easily formed and are often created by the reaction of secondary and tertiary amines, amides, carbamates, and urea derivatives with nitrites or nitrogenous groups.^2^ Since 2018, N-nitrosamine impurities have been a concern in pharmaceutical products due to their toxicity, mutagenicity, and carcinogenicity.

**Figure 1:**
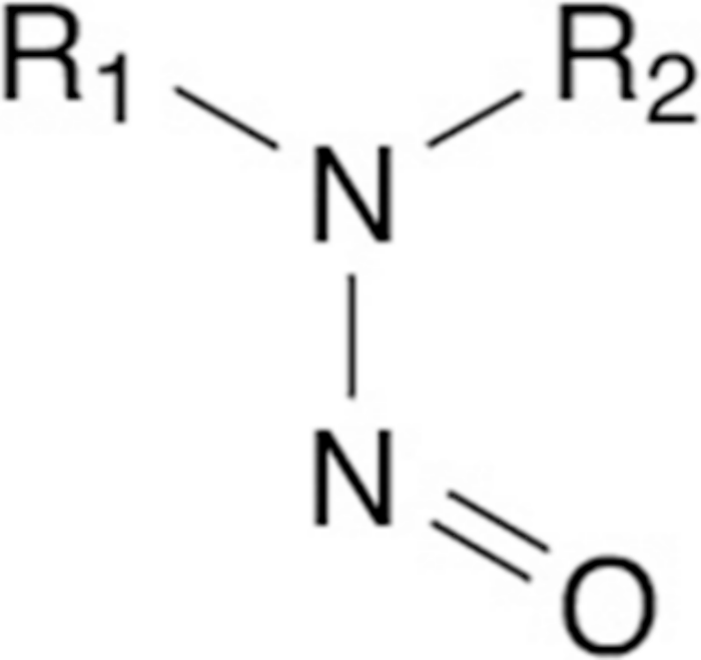
Chemical structure of N-nitrosamine.

## 2 History and Occurrence of N-Nitrosamine Impurities

N-Nitrosamine impurities, a class of chemical compounds, were first described in the chemical literature over 150 years ago (in the 1870s) by Otto Witt.^3^ However, they did not gather much attention until 1956, despite being potential carcinogenic impurities in both human and non-human animals. In 1956, two British scientists, John Barnes and Peter Magee reported that rats and other animals suffer with severe liver damage after the administration of dimethylnitrosamine (NDMA) either orally or parenterally.^4^ N-Nitrosamine impurities were first identified in fish meal, then in cured meats, and at a later stage in beer and malt during the 70s of the twentieth century.^5^

Unexpectedly, contamination with nitrosodimethylamine (NDMA) and related nitrosamine compounds became a problem for drug manufacturers and the Food and Drug Administration (FDA) starting from July 2018. This issue was particularly prominent when NDMA was discovered in certain valsartan products manufactured in China. Subsequently, the recalls were expanded to include other angiotensin II-receptor antagonists (ARBs) such as losartan and irbesartan. Based on these findings, it was suspected that specific synthetic manufacturing processes were responsible for the formation of these N-nitrosamine impurities. As a result, regulatory agencies began working with manufacturers to prevent the presence of N-nitrosamine impurities.^6^ Sartans, including candesartan, irbesartan, losartan, olmesartan, and valsartan, which belong to a class of medicines known as angiotensin II-receptor antagonists, were scrutinized for the presence of dimethylnitrosamine (NDMA). These medicines are primarily used to treat patients with hypertension, heart disease, or kidney diseases.^7^ After the discovery of N-nitrosamine impurities in sartan products, the United States Food and Drug Administration (US FDA) and European Medicines Agency (EMA) announced the presence of a new class of N-nitrosamine impurities in generic active pharmaceutical ingredients (APIs) and drug products. This announcement was followed by numerous product recalls worldwide for sartan products,^8^ leading to extensive investigations.

In early September 2019, regulatory health authorities announced that medicines containing ranitidine were contaminated with unacceptable levels of nitrosodimethylamine impurity.^9^ Following this, countries such as the United States of America,^10^ Canada,^11^ Singapore,^12^ Australia^13^ and Switzerland^14^ recalled and suspended the sale of products containing ranitidine from their markets. This was because ranitidine was the second medication found to contain nitrosodimethylamine impurity, following the sartan products. In December 2019, the United States Food and Drug Administration noted that some metformin products were also contaminated with nitrosodimethylamine impurity.^15^ Additionally, several pharmaceutical companies have received warning letters regarding raw materials, starting materials, and intermediates. These prestage products were also found to contain hazardous N-nitrosamine impurities.^16^

## 3 List of N-Nitrosamine Impurities and Their Limits

Based on a comprehensive literature survey, 13 N-nitrosamine impurities have been identified and reported with their maximum allowable intakes (MAIs).^17^ The list of N-nitrosamine impurities, along with their chemical names, structures, molecular formulas, and maximum allowable intakes, is presented in Table 1. Various regulatory agencies have taken proactive steps by publishing special regulatory guidelines for N-nitrosamine impurities. They have also informed drug manufacturers and healthcare professionals to assess the risk associated with their products available in the market. This is crucial because many N-nitrosamine impurities have the potential to cause cancer in humans due to their toxicity, carcinogenicity, and mutagenicity. According to the ICH (International Council for Harmonization) M7 (R1) guidelines, the maximum daily intake (MDI) of N-nitrosamine impurities is allowed in the range between 26.6 ng/d and 96 ng/d.^18^ This range is applicable only when a single N-nitrosamine impurity is present in an active pharmaceutical ingredient or drug product. However, when multiple N-nitrosamine impurities are present in a single drug or multiple drugs, the overall range (26.6–96 ng/d) must be carefully considered according to the maximum daily dose (MDD) of each individual drug. This consideration is important in order to mitigate the risk of cancer development in patients.

**Table 1:**
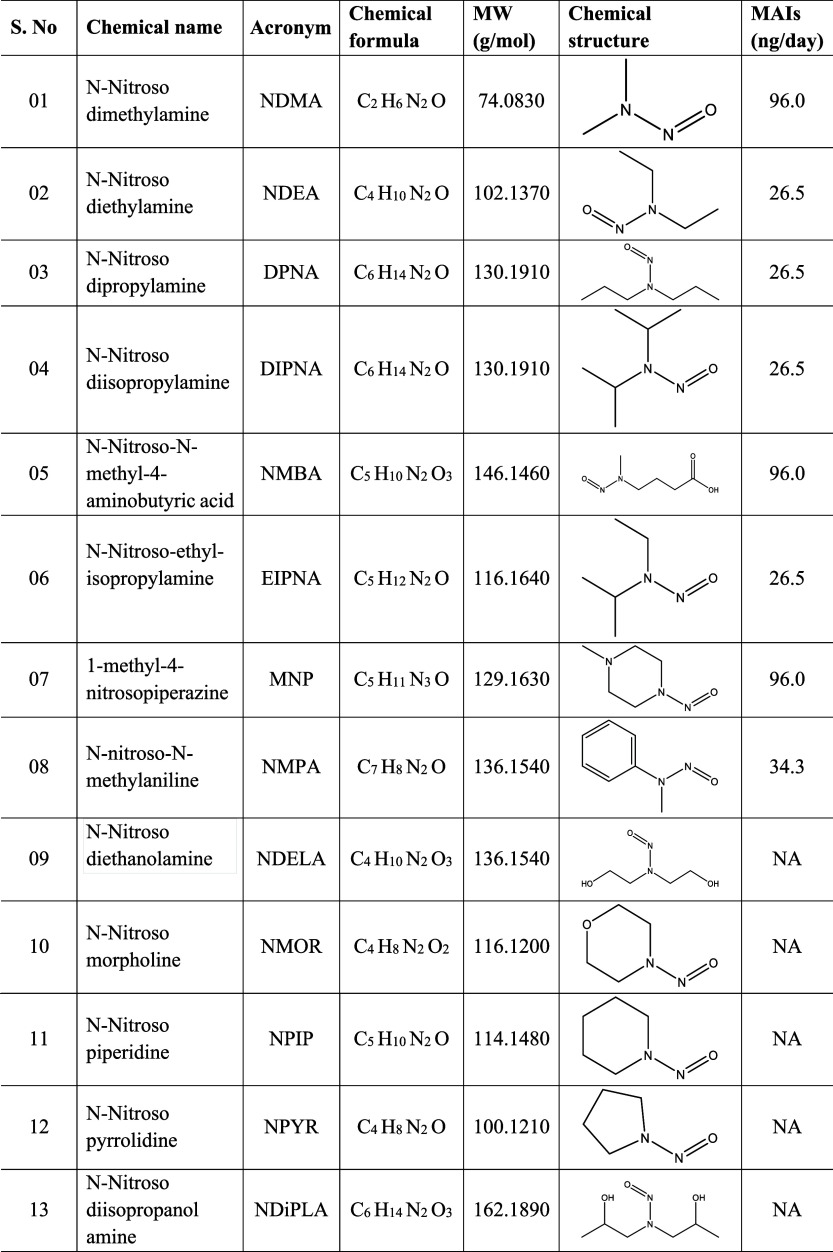
Overview of Chemical Names, Acronyms, Chemical Formulas, Molecular Weights, Chemical Structures and MAIs^a^

a NA = Not available. MW = Molecular weight. MAI = Maximum allowable intake.

## 4 Main Sources of N-Nitrosamine Impurities

Nowadays, humans are exposed to N-nitrosamine impurities in several ways. These include foods such as meat, processed or cooked meat, vegetables, processed vegetables, cereals, milk, dairy or fermented products, pickled foods, spiced foods, cured meat products, processed fish, cocoa, beer, fats, oils, sweets, grains, condiments, cooking oil, margarine, butter, and other beverages including alcoholic ones.^2,19–30^ Exposure can also occur through drinking water, groundwater, treated water, and water treatment plants.^31–72^ Other sources include tobacco (specifically cigarette smoke),^73–92^ pharmaceutical products,^1,2,9,15,93–121^ air pollution,^122–125^ personal care products or cosmetics,^126–129^ rubber packaging materials,^96,130–138^ industrial exposure,^139,140^ and pesticides.^141–144^

Additionally, N-nitrosamine impurities can form during the manufacturing processes of drug substances. This can occur from certain reactive key raw materials, starting materials, intermediates, catalysts, reagents, solvents, and chemicals. These impurities may not be fully removed in the final product.^1,2,97,104,108,110,111,145–150^ Particularly, the use of sodium nitrite (NaNO_2_) or any other nitrate groups can easily form N-nitrosamine impurities during the synthesis of active pharmaceutical ingredients (APIs). Similarly, when using solvents such as N,N-dimethylformamide [DMF], N-methyl pyrrolidone [NMP], N,N-dimethylacetamide [DMA], N-methyl morpholine (NMM), and tributylamine (TBA) during the synthesis process, there is a high probability of forming N-nitrosamine impurities. At times, N-nitrosamine impurities can also be present when contaminated starting materials, raw materials, recovered solvents, reagents, and catalysts are used during the synthesis of active pharmaceutical ingredients. Additionally, when third-party vendors manufacture active pharmaceutical ingredients and related products, they may lack adequate processes and procedures to eliminate N-nitrosamine impurities.

Insufficient optimization of manufacturing processes is another potential source of N-nitrosamine impurities in active pharmaceutical ingredients. These impurities can form under certain reaction conditions such as temperature, pH, and addition of solvents, reagents, raw materials, and intermediates. N-Nitrosamine impurities may also originate from excipients, placebo mixtures, and preservatives used during the drug product formulation process.^151–153^ Similarly, there are numerous other sources that can lead to the formation of N-nitrosamine impurities, including printing operations^64,154^ and the storage of drug substances and drug products under varying temperature and humidity conditions.^155–157^ Furthermore, N-nitrosamine impurities can be produced endogenously, in addition to being introduced exogenously.^158^

## 5 Mechanism of Formation of N-Nitrosamine Impurities

Primarily, two crucial components are needed to form N-nitrosamine impurities in any compound or molecule. These include a nitrosating agent and a secondary or tertiary amine, which are combined under acidic conditions.^17^ A tetrazole ring is formed by using azide-containing reagents such as sodium azide, tributyl azide, and trimethyltin azide. However, these reagents are highly toxic to humans and pose environmental hazards due to their high explosivity. As a result, sodium nitrite is used under acidic conditions to completely eliminate the residual azides. During this process, nitrogen gas and nitrous oxide are released. Furthermore, under acidic conditions, nitrite transforms into nitric acid, acting as a nitrating agent.

Similarly, during the chemical synthesis of certain active pharmaceutical ingredients, solvents such as dimethylformamide, *N*-methyl-2-pyrrolidone, and triethylamine are used. Despite following purification steps, these solvents may leave trace amounts of residue in the final products, which could potentially lead to the formation of N-nitrosamine impurities. Refer to Figure 2 for the mechanism of formation of N-nitrosodimethylamine (NDMA).

**Figure 2:**
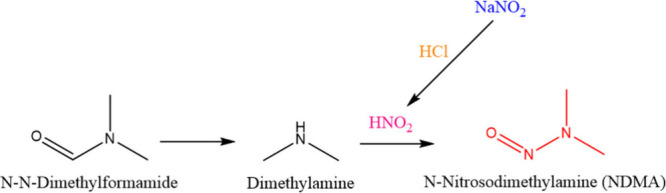
Mechanism of the formation of N-nitrosodimethylamine (NDMA).

N-Nitrosamine impurities can also form due to storage conditions, such as temperature, humidity, and light, in both active pharmaceutical ingredients and drug products. The mechanism of formation of N-nitrosamine impurities due to the storage conditions, nitrosating reagents and secondary amine is shown in Figure 3. Additionally, there are numerous other sources that can lead to the formation of N-nitrosamine impurities, including industrial waste, biological waste, chemical waste, and water disinfection.

**Figure 3:**
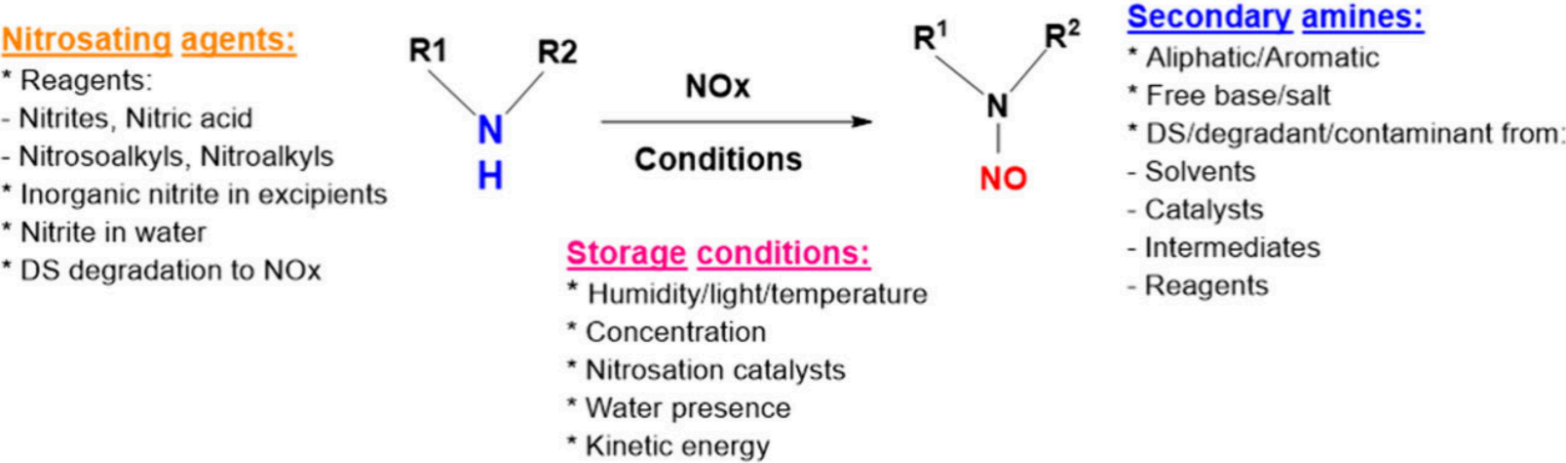
Mechanism of formation of N-nitrosamine impurities due to the storage conditions, nitrosating reagents, and secondary amine.

Also, N-nitrosamines can be formed through a nitrosation reaction between amines and nitrous acid, which is derived from nitrites. This process can be catalyzed by heat and acidic conditions. The formation of N-nitrosamines involves two main steps. In the first step, the nitrite ion (NO_2_^–^) is protonated to form nitrous acid (HNO_2_), which can exist in an equilibrium state with anhydrous dinitrogen trioxide (N_2_O_3_). The latter acts as a nitrosating agent. This reaction proceeds at a faster rate under acidic conditions. In the second step, deprotonation of the amine occurs at a higher pH. Nitrogen oxides (NO_x_), which can be present in water, APIs, excipients, and the atmosphere, can also contribute to the formation of N-nitrosamines. NO_x_ can react with amines to form nitrosamines, especially under acidic conditions. Nitrogen oxides (NO_x_), specifically nitrogen dioxide (NO_2_) and nitric oxide (NO), can also contribute to the formation of nitrites. When these nitrogen oxides are released into the atmosphere as air pollutants, they dissolve in rainwater and form nitric acid (HNO_3_). Nitric acid can further react with other compounds in the environment and eventually convert to nitrite (NO_2_^–^) in water. Essentially, nitrites and NO_x_ are significant contributors to the formation of N-nitrosamine impurities in drugs.

## 6 Pharmaceutical Products Containing N-Nitrosamine Impurities

The names and structures of pharmaceutical products containing N-nitrosamine impurities are listed in Figures 4 and 5. Furthermore, the names of the products and their therapeutic areas/classes that contain N-nitrosamine impurities, along with treatment details, are reported in Table 2.

**Figure 4:**
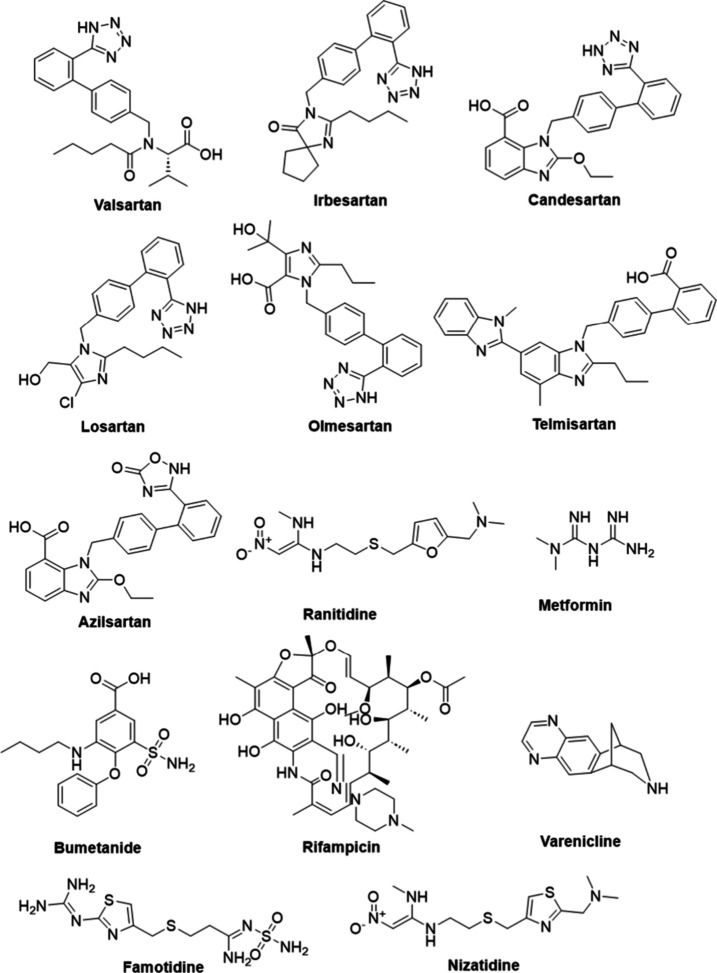
Names and structures of products containing N-nitrosamine impurities

**Figure 5:**
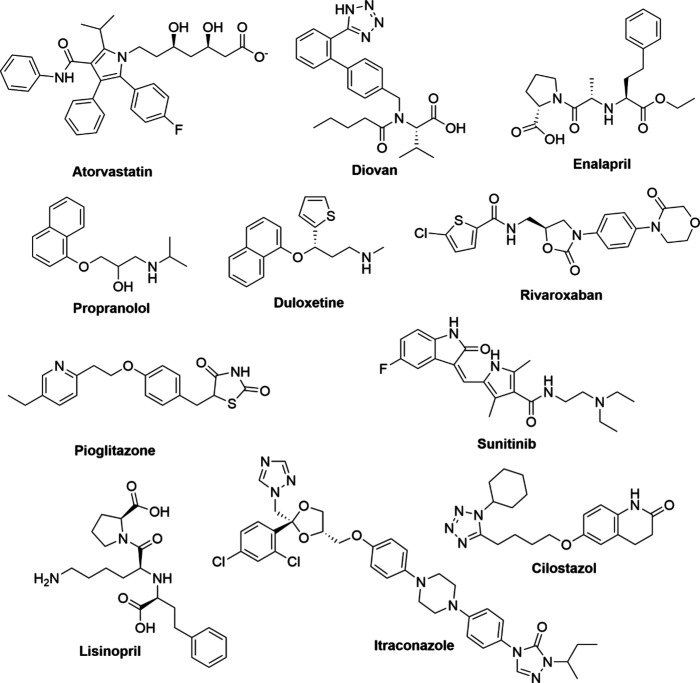
Names and structures of products containing N-nitrosamine impurities.

**Table 2:** Product Names and Therapeutic Areas/Classes That Contain N-Nitrosamine Impurities, Including Treatment Details

S. No.	Product name	Therapeutic area/Class	Used for (treatment)	Remarks
01	Sartans (azilsartan, valsartan, telmisartan, olmesartan, losartan and irbesartan)	Angiotensin II receptor blockers (ARBs)	Hypertension (high blood pressure)	Previous products containing N-nitrosamine impurities
02	Metformin hydrochloride	Type 2 diabetes mellitus	Control the amount of glucose (sugar) in your blood	
03	Ranitidine hydrochloride	H2 blockers	Indigestion, heartburn and acid reflux, gastro-esophageal reflux disease	
04	Rifampicin	Antimycobacterials	Diverse mycobacterial infections and gram-positive bacterial infections	Recent products containing N-nitrosamine impurities
05	Champix	Tobacco use cessation	Smoking cessation and for the treatment of dry eye disease	
06	Famotidine	Histamine H2 receptor antagonist	Stomach ulcers (gastric and duodenal), erosive esophagitis (heartburn or acid indigestion), and gastroesophageal reflux disease (GERD)	
07	Nizatidine	Histamine H2 antagonists	stomach ulcers (active benign gastric ulcer or duodenal ulcer), erosive and ulcerative esophagitis (heartburn or acid indigestion), and gastroesophageal reflux disease (GERD)	
08	Atorvastatin calcium	HMG-CoA reductase inhibitors (statins)	Cholesterol lowering agent (statin)	
09	Bumetanide	Loop diuretics or ″water pills (edema)	Treatment of edema associated with congestive heart failure, hepatic and renal disease	
10	Itraconazole	Antifungal	Oropharyngeal or esophageal candidiasis (thrush, oral thrush)	
11	Diovan	Angiotensin II receptor blockers (ARBs)	High blood pressure and heart failure	
12	Enalapril maleate	ACE inhibitor medication	High blood pressure (hypertension)	
13	Propranolol	Beta-blocker	High blood pressure	
14	Lisinopril	ACE inhibitors	High blood pressure (hypertension)	
15	Duloxetine	Selective serotonin and norepinephrine reuptake inhibitors (SNRIs)	Depression and anxiety	
16	Rivaroxaban	Anticoagulant medicine	Deep vein thrombosis and pulmonary emboli and prevent blood clots in atrial fibrillation and following hip or knee surgery	
17	Pioglitazones	Oral antidiabetic/Targeting insulin resistance	High blood sugar levels caused by type 2 diabetes	
18	Glifizones	Oral antidiabetic/Targeting insulin resistance	High blood sugar levels caused by type 2 diabetes	
19	Cilostazol	Platelet-aggregation inhibitors (antiplatelet medications)	Intermittent claudication due to peripheral vascular disease	
20	Sunitinib malate	Kinase inhibitors	Gastrointestinal stromal tumors	

## 7 List of Pharmaceutical Products Recalled from the Market Due to the Presence of N-Nitrosamine Impurities

In July and August 2018, the United States Food and Drug Administration initiated the first recall of a generic medicine called valsartan. This recall was due to the discovery of an impurity, N-nitrosodimethylamine, which is a potential human carcinogen. The active pharmaceutical ingredient in valsartan was manufactured by a Chinese company named Zhejiang Huahai. Subsequently, another N-nitrosamine impurity, nitrosodiethylamine, was found in an angiotensin receptor blocker called losartan, produced by Hetero Lab Limited. Interestingly, the active pharmaceutical ingredient in losartan was also manufactured by the same chinese company, Zhejiang Huahai.^120,159^ Principally, valsartan and losartan are generic medicines that are prescribed as angiotensin receptor blockers to treat high blood pressure in patients.

Subsequently, other regulatory agencies, such as the European Medicines Agency and the European Directorate for the Quality of Medicines and HealthCare (EDQM), also recalled both valsartan and losartan products. Specifically, the United States Food and Drug Administration recalled angiotensin II receptor blocker medicines, which included valsartan, losartan, and irbesartan, from approximately 16 drug manufacturers (Aurobindo Pharma USA, Inc. (Acetris), Teva/Actavis & Prinston/Solco, Camber Pharmaceuticals, Inc., Heritage Pharmaceuticals Inc. (Vivimed), Lupin Pharmaceuticals Inc., Macleods Pharmaceutical Ltd., Mylan Pharmaceuticals, Inc., PD-Rx Pharmaceuticals Inc, Remedyrepack, Inc. (Hetero/Camber), Torrent Pharmaceuticals Limited, etc.) for many lots/batches from the market.^160^ Furthermore, a comprehensive investigation was conducted, including a risk assessment for all sartan generic medicines for the presence of N-nitrosamine impurities.

In early 2020, the United States Food and Drug Administration voluntarily recalled products from several companies due to the presence of N-nitrosodimethylamine in the metformin drug product.^161,162^ In April 2020, the same agency announced the immediate withdrawal of over-the-counter (OTC) ranitidine drugs, commonly known by the brand name Zantac, from the market due to the presence of a carcinogenic contaminant called N-nitrosodimethylamine. In September 2021, Pfizer, one of the largest pharmaceutical manufacturing companies, voluntarily recalled all lots/batches of Chantix 0.5 mg and 1 mg tablets from consumers due to the presence of N-nitrosamine and N-nitroso-varenicline, which were above the interim acceptable intake limits proposed by the Food and Drug Administration.^163^

## 8 Regulatory Guidelines for N-Nitrosamine Impurities

Regulatory agencies have introduced a three-step process or guidance. This process must be followed by drug manufacturers and applicants to mitigate the presence of unsafe N-nitrosamine impurities in their drug products. The steps are as follows:

1. Conduct risk assessments for N-nitrosamine impurities in their active pharmaceutical ingredients and drug products.
2. Conduct confirmatory testing if risks are identified.
3. Report them if noted.

After detection of N-nitrosamine impurities in sartan products, the United States Food and Drug Administration (US FDA) initiated significant efforts. They published guidelines for drug manufacturers and applicants that also apply to over-the-counter medicines. These guidelines offer recommendations and define acceptable intake levels for predicting, identifying, and confirming potential carcinogenic and mutagenic N-nitrosamine impurities present in drug substances and their related impurities (NDSRIs). These guidelines are then applied to the final drug products.^164^ Subsequently, the FDA implemented additional guidelines titled “control of nitrosamine impurities in human drugs” and “recommended acceptable intake limits for nitrosamine drug substance-related impurities (NDSRIs)”. These serve as potential control strategies and provide an effective evaluation framework for drug substance-related N-nitrosamine impurities.^165,166^ The FDA is continuously working on, updating, and implementing many more guidelines to avoid and control these N-nitrosamine impurities with patient safety and risk mitigation as the primary considerations.

The European Medicines Agency has assessed the risk of formation or presence of N-nitrosamine impurities during the manufacturing of medicines. It has provided guidance to marketing authorization holders on how to avoid the presence of N-nitrosamine impurities.^120,167,168^ In July 2023, the European Medicines Agency updated and amended the guidelines to include the “carcinogenic potency categorization approach” (CPCA) and the “enhanced Ames test” (EAT) for establishing the acceptable intakes (AIs) for N-nitrosamine impurities.

The European Directorate for the Quality of Medicines & HealthCare has also implemented and updated the guidelines. These include new scientific approaches for the categorization of N-nitrosamine impurities and the establishment of acceptable intakes. Additionally, a separate Appendix lists the N-nitrosamine impurities for which acceptable intakes have been established by the EMA nonclinical working party (NcWP).^169,170^

N-Nitrosamine impurities are classified as “class 1 impurities” and “known mutagenic carcinogens” by the ICH M7­(R1) guideline, based on both carcinogenicity and mutagenicity data. The “International Agency for Research on Cancer” (IARC) categorizes N-nitrosamine impurities as 2A probable carcinogens, based on available data from numerous studied species.^171–176^ The United States Pharmacopeia (USP) has implemented the general chapter 1469 for N-nitrosamine impurities, aligning with the ICH M7­(R1) guidelines.^177^ Additionally, the government of Canada has issued regulatory guidelines to applicants and market authorization holders (MAHs) on evaluating and managing the risks of N-nitrosamine impurities in humans when using pharmaceutical, biological, and radiopharmaceutical products.^178,179^

The Brazilian health authority, ANVISA, has released a regulatory guide on the control of N-nitrosamine impurities in active pharmaceutical ingredients (APIs) and medicines, as detailed in “ANVISA - Brazil released a new guidance on nitrosamines - vina GMP (Good Manufacturing Practices)”.^180^ Additionally, the China National Medical Products Administration issued technical guidelines for the study of N-nitrosamine impurities in chemical drugs on May 8, 2020. These guidelines facilitated discussions about the sources of N-nitrosamine impurities in drug products and strategies for their control. The control strategy was outlined from various perspectives, including the basic concept of control, control limits, the establishment of analytical methods, and risk control throughout the drug product lifecycle.^181^

In addition to the above-mentioned major regulatory agencies, other regulatory agencies such as the Therapeutic Goods Administration (TGA), an Australian regulatory agency,^182^ the Medicines and Healthcare Products Regulatory Agency (MHRA), a European regulatory agency, and many other agencies are closely working with other international regulators and medicine sponsors to investigate and address N-nitrosamine impurity issues in medicines.^183^

## 9 Available Analytical Methods for N-Nitrosamine Impurities Determination

Considering the maximum daily dose and the threshold of toxicological concern (TTC), N-nitrosamine impurities have low specification and quantification limits. As a result, the analytical methods under development should be able to detect and quantify them with high sensitivity, selectivity, precision, and accuracy. However, these requirements may not be achievable with traditional methods developed using standard analytical techniques such as high-performance liquid chromatography (HPLC), ultraperformance liquid chromatography (UPLC), gas chromatography (GC), and ultraviolet spectroscopy. Therefore, analytical methods need to be developed using advanced analytical techniques, such as liquid chromatography mass spectrometry (LC-MS), gas chromatography mass spectrometry (GC-MS), and capillary electrophoresis (CE) to accurately detect and quantify N-nitrosamine-related impurities in pharmaceutical products. The reality is that due to the lack of sensitive analytical methods, N-nitrosodimethylamine was not detected in the valsartan active pharmaceutical ingredient manufactured by a Chinese company called Zhejiang Hua Hai. Otherwise, product recalls would not have been necessary. Based on this incident, various regulatory agencies worldwide have increased their focus on N-nitrosamine impurities since 2018. Consequently, drug manufacturers have started developing sensitive and selective analytical methods, using advanced techniques to detect the presence of N-nitrosamine impurities.

Based on a comprehensive literature survey, it is evident that numerous sensitive and selective analytical methods have been developed, validated, and reported by various regulatory agencies and drug manufacturers. These methods address the detection of N-nitrosamine impurities in active pharmaceutical ingredients, drug products, bioanalytical products, water, tobacco, and other matrices. Various analytical techniques have been employed, including fast liquid chromatography (fast-LC), liquid chromatography mass spectrometry, gas chromatography mass spectrometry, capillary electrophoresis, and a few other techniques.

The United States Food and Drug Administration developed and published liquid chromatography high resolution mass spectrometry (LC-HRMS) methods for eight N-nitrosamine impurities for detection and quantification in several pharmaceutical drugs (valsartan, losartan, other ARBs, ranitidine, metformin, chloroquine and hydroxy chloroquine).^184,185^ Also, the United States Food and Drug Administration has published a general chapter 1469 about the control of six N-nitrosamine impurities (NDMA, NDEA, NEIPA, NDIPA, NMBA and NDBA) wherein there were four analytical methods reported such as liquid chromatography-high resolution mass spectrometry, headspace-gas chromatography-mass spectrometry (GC-HS-MS/MS), liquid chromatography with tandem mass spectrometry (LC-MS/MS), and gas chromatography-tandem mass spectrometry (GC-MS/MS).^186^

The European Pharmacopoeia (Ph. Eur.) commission has implemented a new general chapter for the analysis of N-nitrosamine impurities in active substances wherein there were three procedures reported using three (GC-MS, LC-MS/MS and GC-MS/MS) sophisticated instruments; these three procedures cover a total of seven N-nitrosamine impurities: N-nitroso-dimethylamine (NDMA), N-nitroso-diethylamine (NDEA), N-nitroso-dibutylamine (NDBA), N-nitroso-N-methyl-4-aminobutyric acid (NMBA), N-nitroso-diisopropylamine (NDiPA), N-nitroso-ethyl-isopropylamine (NEiPA) and N-nitroso-dipropylamine (NDPA).^187^

The Official Medicines Control Laboratory (OMCL) at SWISS MEDIC developed and reported a new method using gas chromatography mass spectrometry with multiple reaction monitoring (MRM) for the determination of six N-nitrosamine impurities (NDMA, NDEA, EIPNA, DIPNA, DPNA and DBNA) in both drug substances and drug products of losartan, valsartan, irbesartan, candesartan and olmesartan.^188^

The Taiwan Food and Drug Administration developed and published a couple of methods for successful determination of N-nitrosamine impurities in sartan drug substances and drug products (candesartan, irbesartan, losartan, olmesartan, telmisartan, and valsartan) using liquid chromatography-tandem mass spectrometry and gas chromatography-mass spectrometry.^189,190^

Germany’s Official Medicines Control Laboratory (OMCL) developed and reported the first efficient method for simultaneous analysis of N-nitroso-dimethylamine and N-nitroso-diethylamine in sartan products for both film-coated tablets and drug substances having a lower limit of detection (LOD) and limit of quantification (LOQ) using ultra high-performance liquid chromatography-mass spectrometry (UHPLC-APCI-MS/MS) with a multiple reaction monitoring (MRM) mode.^191^

In addition to the regulatory agencies mentioned above, analytical instrument manufacturers/vendors such as Agilent Technologies, Waters, and Shimadzu have developed and published several analytical methods. They utilized their own sensitive instruments for liquid chromatography-mass spectrometry and gas chromatography-mass spectrometry to detect various N-nitrosamine impurities in numerous medicines.

Agilent technologies developed and reported a new method for the analysis of five N-nitrosamine impurities (NDMA, NDEA, NEIPA, NDIPA and NDBA) in drug products and drug substances using Agilent gas chromatography-mass spectrometry instrumentation.^192^ Similarly, they also developed a couple of new gas chromatography-mass spectrometry methods and liquid chromatography-mass spectrometry methods for the screening and analysis of N-nitrosamine impurities in sartan drug products and drug substances.^193–196^ Agilent also developed and published a new liquid chromatography-mass spectrometry method for simultaneous determination of eight N-nitrosamine impurities in metformin extended-release tablets and N-nitroso-dimethylamine impurity in ranitidine using an Agilent 6470 triple quadrupole liquid chromatographer-mass spectrometer.^197,198^

The Waters technologies developed and reported a new high sensitivity liquid chromatography-mass spectrometry method for the quantitation of six N-nitrosamine genotoxic impurities (NDMA, NDEA, NDBA, NMBA, NEIPA and NDIPA) in ranitidine drug products using the Waters ACQUITY UPLC I-Class/Xevo TQ-XS tandem quadrupole mass spectrometer.^199^

Shimadzu established and reported a few sensitive and selective gas chromatography-mass spectrometry methods and liquid chromatography-mass spectrometry methods as per United States Pharmacopeia general chapter 1469 for various N-nitrosamine impurities in several drug substances and drug products such as sartan products, metformin and ranitidine.^200–204^

In addition to the regulatory agencies mentioned above and analytical instrument manufacturers/vendors, drug manufacturers and researchers have also developed and published various methods. These methods are used to detect N-nitrosamine impurities in numerous medicines, utilizing analytical techniques such as high-performance liquid chromatography, ultraperformance liquid chromatography, liquid chromatography–mass spectrometry, gas chromatography–mass spectrometry, supercritical fluid chromatography (SFC), and capillary electrophoresis.

The National Institute of Health (NIH) developed and validated a new method for four N-nitrosamine impurities (NDMA - N,N-dimethylnitrous amide, NDEA - N,N-diethylnitrous amide, NMBA - 4-[methyl­(nitroso)­amino]­butanoic acid and NEIPA - N-ethylpropan-2-amine) in valsartan, losartan and irbesartan drugs using high-performance liquid chromatography mass spectrometry (HPLC-MS/MS) with an ionization source called atmospheric pressure chemical ionization (APCI).^205^

Numerous high-performance liquid chromatography methods have been developed and documented by various researchers. These methods are used for the detection of different N-nitrosamine impurities in several drugs, which include atorvastatin, itraconazole,^206^ losartan, valsartan,^207^ diovan, losartan,^208^ enalapril maleate,^209^ losartan,^210^ propranolol,^211^ valsartan^212^ and lisinopril.^213^ In addition to the above drugs, high-performance liquid chromatography methods were also developed and reported for estimation of N-nitrosamine impurities in water,^214^ cigarettes^215^ and food.^216^

Several liquid chromatography–mass spectrometry methods have been developed and reported by several researchers for quantification of various N-nitrosamine impurities in many drugs, including ranitidine (a bioanalytical method),^217^ losartan, valsartan,^218^ sartan drugs,^219^ valsartan,^220^ ranitidine,^221^ rifampicin,^222^ metformin,^223^ telmisartan,^224^ sartans (azilsartan, valsartan, telmisartan, olmesartan, losartan and irbesartan),^225^ valsartan,^226^ ranitidine,^227^ duloxetine,^228^ valsartan and irbesartan,^229^ olmesartan,^230^ rifampicin,^231^ metformin,^232^ sartans,^233^ sartans,^234^ metformin,^235^ valsartan,^236^ rivaroxaban,^237^ metformin^238^ and valsartan.^239^ Additionally, liquid chromatography-mass spectrometry methods were also developed and reported for estimation of N-nitrosamine impurities in cigarette tobacco, cigar tobacco and smokeless tobacco,^240^ tobacco and mainstream cigarette smoke,^241^ tobacco-specific N-nitrosamine impurities,^242^ biopharmaceuticals,^243^ groundwater^244^ and wastewater.^245^

Many gas chromatography-mass spectrometry methods have been developed and reported by several researchers for quantification of various N-nitrosamine impurities in various drugs, including metformin, ranitidine,^246^ valsartan,^247^ metformin,^248^ sartans, ranitidine, metformin,^249^ ranitidine products,^250^ cilostazol, sunitinib malate, olmesartan medoxomil,^251^ metformin,^252^ sartan substances,^253^ sartan pharmaceuticals,^190^ ranitidine, metformin, nizatidine^254^ and losartan.^255^ Additionally, gas chromatography-mass spectrometry methods were also developed and reported for estimation of N-nitrosamine impurities in pesticides,^142^ water,^256^ rat feces^257^ and children’s products.^258^

In addition to the HPLC, UPLC, LC-MS, and GC-MS methods mentioned above, several other techniques have also been developed and published by various researchers. These techniques are also used for detecting N-nitrosamine impurities, such as supercritical fluid chromatography for valsartan, losartan, metformin, ranitidine and pioglitazone products,^259^ an another supercritical fluid chromatography method for valsartan, losartan and sartan related impurities,^260^ a high throughput automated microsolid phase extraction MS/MS method for generic losartan, valsartan, olmesartan, irbesartan, telmisartan drug substances and drug products^261^ and a capillary electrophoresis electrospray ionization (ESI) mass spectrometry method for tobacco specific N-nitrosamine impurities in rabbit serum (CE-ESI/MS).^262^ An overview of current analytical methods that were developed for the determination of N-nitrosamine impurities is presented in Table 3.

**Table 3:** Overview of Current Analytical Methods for the Determination of N-Nitrosamine Impurities^b^

Product name	Technique	Title of the method	N-Nitrosamine impurity	LOD (ppm)	LOQ (ppm)	Superiority/Advantages	Ref
Valsartan, losartan, and other ARBs	LC-HRMS	LC-HRMS based analytical platform to determine nitrosamines in pharmaceuticals: modern analytical techniques meet regulatory needs	NDMA, NDEA, NEIPA, NDIPA, NDBA, NMBA	0.003–0.01	0.05	High sensitivity, reliability and capable of detecting and quantitating eight nitrosamine impurities in various drug products	^184^
Ranitidine	LC-HRMS	LC-HRMS based analytical platform to determine nitrosamines in pharmaceuticals: modern analytical techniques meet regulatory needs	NDMA	0.01	0.03		^184^
Metformin	LC-HRMS	LC-HRMS based analytical platform to determine nitrosamines in pharmaceuticals: modern analytical techniques meet regulatory needs	NDMA	0.01	0.03		^184^
Metformin	LC-HRMS	LC-HRMS based analytical platform to determine nitrosamines in pharmaceuticals: modern analytical techniques meet regulatory needs	NDMA, NDEA, NEIPA, NDIPA, NDPA, NDBA NMPA, NMBA	0.001–0.005	0.005–0.02		^184^
Chloroquine & hydroxy chloroquine	LC-HRMS	LC-HRMS based analytical platform to determine nitrosamines in pharmaceuticals: modern analytical techniques meet regulatory needs	NDMA, NDEA, NEIPA, NDIPA, NDPA, NDBA NMPA, NMBA	0.003–0.006	0.02		^184^
Metformin	LC-ESI-HRMS	LC-ESI-HRMS method for the determination of nitrosamine impurities in metformin drug substance and drug product	NDMA	0.005	0.01	High sensitivity, accuracy and capable of detection and quantification of 8 nitrosamines	^185^
			NDEA	0.002	0.02		
			NEIPA	0.003	0.02		
			NDIPA	0.001	0.02		
			NDPA	0.001	0.005		
			NMPA	0.001	0.005		
			NDBA	0.001	0.005		
			NMBA	0.002	0.005		
NA	LC-HRMS	⟨1469⟩ Nitrosamine Impurities^a^	NDMA, NDEA, NEIPA, NDIPA, NMBA, NDBA	NA	0.05	Sensitivity, selectivity, quantitative and qualitative procedures for testing of nitrosamines	^186^
	GC-HS-MS/MS		NDMA, NDEA, NEIPA, NDIPA		0.02		
	LC–MS/MS		NDMA, NDEA, NEIPA, NDIPA, NMBA, NDBA		0.01 and 0.02		
	GC-MS/MS		NDMA, NDEA, NEIPA, NDIPA, NDBA		0.005		
NA	GC-MS, LC-MS & GC-MS/MS	Ph. Eur. Commission adopts a new general chapter for the analysis of N-nitrosamine impurities	NDMA, NDEA, NDBA, NMBA, NDIPA, NEIPA, NDPA	NA	NA	Useful as limit test and/or quantitative test	^187^
Sartans	GC-MS/MS	Swiss medic limit test for the determination of nitrosamines by GC-MS/MS^a^	NDMA, NDEA, EIPNA, DIPNA, DPNA, DBNA	NA	15 ppb	Sensitivity, selectivity and suitable for the determination of 6 nitrosamines at 15 ppb (LOQ)	^188^
Sartans	LC-MS/MS	A multianalyte LC-MS/MS method for screening and quantification of nitrosamines in sartans	NDMA, NMEA, NDEA, NEIPA, NDiPA, NDPA, NDiBA, NDBA, NPIP, NMOR, NDiNA, NDCHA, NDPhA	20 ng/g	50 ng/g	Sensitivity, selectivity and suitable for screening, determination and quantification of 12 nitrosamines	^189^
Sartans	GC-MS/MS	Screening of nitrosamine impurities in sartan pharmaceuticals by GC-MS/MS	NDMA, NMEA, NDEA, NEIPA, NDiPA, NDPA, NDiBA, NDBA, NPIP, NMOR, NDiNA, NDCHA, NDPhA	15–250 ng/g	50–250 ng/g	Sensitivity, selectivity, useful for monitoring and determining 13 nitrosamines as well as quality monitoring purposes	^190^
Sartans	LC-MS/MS	Test method for the determination of NDMA and NDEA by LC-MS/MS in sartan containing film coated tablets	NDMA	0.08	0.2	Sensitivity, selectivity and useful for detection and quantitative determination	^191^
			NDEA	0.02	0.04		
Sartans	GC/MS/MS	Analysis of five nitrosamine impurities in drug products and drug substances using agilent GC/MS/MS instrumentation^a^	NDMA, NDEA, NEIPA, NDIPA, NDBA	NA	0.0025, 0.0005, 0.00025, 0.0025, 0.008	High sensitivity with improved LOQs and reliable quantification of 5 residues	^192^
Sartans	GC/MS/MS	Screening of nitrosamine impurities in drug products and drug substances using agilent GC/MS/MS instrumentation	NDMA, NDEA, NMOR, NMEA, NPYR, NPIP, NEIPA, NDIPA, NDPA, NDBA, NMPA, NMPEA, NDPh	0.05 – 2 ppb	1 – 10 ppb	Reliable screening of 13 nitrosamines at trace level with good resolution and lower detection limits	^193^
Sartans	GC-TQ	Quantification of nine nitrosamine impurities in sartan drugs using an agilent GC-TQ	NDEA, NEIPA, NDIPA, NDMA, NDPA, NDBA, NPIP, NMEA, NPYR	NA	0.0006, 0.0006, 0.0006, 0.001, 0.001, 0.001, 0.001, 0.02, 0.02	Reliable quantification of 9 nitrosamines at trace level with lower detection limits	^194^
Sartans	LC/MS	Determination of nitrosamine impurities using the ultivo triple quadrupole LC/MS	NDMA, NDEA, NMBA, NEIPA, NDIPA, NDBA, NMEA, NPyR, NPIP, NMPhA, NMIPA, N-*tert*-butyl-N-ethyl nitrosamine	0.05, 0.025, 0.05, 0.025, 0.025, 0.05, 0.075, 0.075, 0.1, 0.075, 0.025, 0.075 ng/mL	0.1, 0.05, 0.1, 0.05, 0.05, 0.1, 0.1, 0.1, 0.15, 0.1, 0.05, 0.1 ng/mL	Sensitivity, detection and quantification of low concentration levels	^195^
Sartans	LC-MS/MS	Determination of nitrosamine impurities using the high-resolution agilent 6546 LC/Q-TOF	NDMA, NDEA, NMBA, NEIPA, NDIPA, NDBA, NMEA, NPyR, NPIP, NMPhA, NMIPA	0.1, 0.05, 0.25, 0.1, 0.075, 0.1, 0.05, 0.1, 0.075, 0.25, 0.075 ng/mL	0.25, 0.1, 0.5, 0.25, 0.15, 0.25, 0.1, 0.15, 0.1, 0.5, 0.1 ng/mL	Sensitivity and quantification at low concentration levels	^196^
Metformin	LC/MS	Simultaneous determination of eight nitrosamine impurities in metformin using the agilent 6470 triple quadrupole LC/MS	NDMA, NDEA, NEIPA, NDIPA, NMBA, NDPA, NMPA, NDBA	0.002, 0.002, 0.002, 0.002, 0.001, 0.001, 0.001, 0.001 ng/mL	0.01, 0.01, 0.01, 0.01, 0.005, 0.005, 0.005, 0.005 ng/mL	Reproducibility, sensitivity and detection of 8 nitrosamines at low concentration levels	^197^
Ranitidine	LC/MS	Determination of NDMA impurity in ranitidine using the agilent 6470 triple quadrupole LC/MS	NDMA	0.1 ng/mL	0.25 ng/mL	Highly sensitive, very reproducible and diverter valve program can be used to exclude the API	^198^
Ranitidine	LC-MS	High sensitivity quantitation of nitrosamine genotoxic impurities: LC-MS analysis of ranitidine drug product using the waters ACQUITY UPLC I-Class/Xevo TQ-XS tandem quadrupole mass spectrometer	NDMA, NDEA, NDBA, NMBA, NEIPA, NDIPA	NA	0.025–0.1 ng/mL	Highly sensitive, accurate, simple and reproducible method for detection and quantification of multiple nitrosamine impurities	^199^
Metformin	LC-MS	Got DMF? Chromatographic separation and identification of NDMA and DMF using LCMS-9030	NDMA	NA	NA	Sensitive and selective method for identification and quantification	^200^
Sartans	GC-MS	Analysis of N-nitrosodimethylamine (NDMA) & N-nitrosodiethylamine (NDEA) in pharmaceutical substance by HSGCMS/MS	NDMA, NDEA	NA	2.5 ppb	Sensitive, selective, fast, reproducible, reliable, accurate and linear method for trance level quantification	^201^
Metformin	LC-MS	Simultaneous analysis of nitrosamines impurities in metformin drug substance and drug product using shimadzu LCMS-8050 triple quadrupole mass spectrometer	NDMA, NDEA, NEIPA, NDIPA, NDPA, NMPA, NDBA, NMBA	NA	1, 0.3, 0.5, 0.5, 0.3, 0.5, 0.3, 0.3 ng/mL	Robust, reliable, sensitive and specific method for identification and quantitation of 8 nitrosamines	^202^
Metformin	GC-MS/MS	Quantitation of 5 NSA in metformin API as per proposed USP General Chapter ⟨1469⟩ Procedure-4 by GC-MS/MS	NDMA, NDEA, NEIPA, NDIPA, NDBA, NMBA	0.1 ppb	0.25 ppb	Trace level quantitation	^203^
Sartans	GC-MS/MS	Determination of nitrosamine impurities in sartan drug products by GC-MS/MS method	NDMA, NMEA, NDBA, NDEA, NDPA, NPYR, NPIP	NA	NA	Highly sensitive and reliable method for the analysis of 7 nitrosamines	^204^
Sartans	HPLC-MS/MS	Development and validation of four nitrosamine impurities determination method in medicines of valsartan, losartan, and irbesartan with HPLC-MS/MS (APCI)	NDMA, NDEA, NMBA, NEIPA	0.2 ng/mL	0.4 ng/mL	Higher sensitivity, selectivity and can be used as routine quality control method	^205^
Atorvastatin & Itraconazole	LC-UV	Formic acid-aided sample preparation method for sensitive and simultaneous analysis of eight nitrosamines in poorly water-soluble pharmaceutical drugs using liquid chromatography-ultraviolet detection	NDMA, NMEA, NDEA, NEIPA, NDPA, NDIPA, NDBA, NMBA	NA	NA	Specific, sensitive detection and quantification method for simultaneous determination of 8 nitrosamines	^206^
Valsartan & Losartan	HPLC	Cost-effective, green HPLC determination of losartan, valsartan and their nitrosodiethylamine impurity: application to pharmaceutical dosage forms	NDEA	0.2	0.5	Simple, fast, green, cost-effective and lowest ecological impact method	^207^
Sartans	HPLC	Analysis of nitrosamines using unique stationary phase technology	NDMA, NMOR, NMEA, NPPYR, NDEA, NPIP, NDPA, NDBA, NDPHA	NA	NA	Simultaneous detection of 9 nitrosamines with unique stationary phase technology and potential solvent residues	^208^
Enalapril maleate	HPLC-FD	Development and validation of a method for the semiquantitative determination of n-nitrosamines in active pharmaceutical ingredient enalapril maleate by means of derivatization and detection by HPLC with fluorimetric detector	NDMA, NDEA	0.013 and 0.017	0.038 and 0.050	Reproducibility, sensitivity and specificity	^209^
Losartan	HPLC-UV	Quantification and Validation of a HPLC-UV method for simultaneous analysis of nitrosamine impurities (NDMA, NDEA and NDIPA) in losartan	NDMA, NDEA, NDIPA	0.011 0.017 0.018	0.021 0.025 0.028	Simple, easily adoptable, specific, linear, precise and accurate method	^210^
Propranolol	LC-MS	Sensitive and reproducible quantification of N-nitroso­propranolol in a propranolol drug substance and product	N-nitroso propranolol impurity	0.005 ng/mL	0.010 ng/mL	Simple, accurate, highly reproducible and low-level quantification	^211^
Valsartan	HPLC	Rapid and efficient high-performance liquid chromatography analysis of N-nitrosodimethylamine impurity in valsartan drug substance and its products	NDMA	0.0085	0.0285	Rapid, efficient and useful in quality control for the APIs and drug products routine analysis	^212^
Lisinopril	HPLC-FLD	Development and validation of an HPLC-FLD method for the determination of NDMA and NDEA nitrosamines in lisinopril using precolumn denitrosation and derivatization procedure	NDMA, NDEA	4.7 ng/mL & 0.04 μg/mL	14.4 ng/mL & 0.13 μg/mL	Simple, reliable, selective, sensitive and alternative method	^213^
Water	HPLC-PCUV	Analysis of N-nitrosamines and other nitro(so) compounds in water by high-performance liquid chromatography with postcolumn UV photolysis/Griess reaction	N-Nitroso NDELA, NDMA, NDEA, NMOR, NPYR, NDEA, NPIP, NDPA, NDBA, N-Nitro DMNA	4–28 ng/L	NA	Novel and powerful screening tool for known and other nitro (so) compounds	^214^
Cigarette smoke	HPLC	HPLC analysis and reactions of N-nitrosamines	NDMA, NDEA, NDPA, NDPhA	Up to 20 mg/L	NA	Simple, rapid, accurate, qualitative or trace quantitative method with multiple uses	^215^
Food	HPLC-UV-FLD	Analytical methods studies on a novel method for the determination of nitrosamines in food by HPLC-UV-FLD coupling with terbium-doped carbon dots	NDMA, NMor, NPYR, NDEA, NPIP	2.25 μg/L	NA	Novel, selective, sensitive and online HPLC-UV-FLD approach for the determination of 5 kinds of nitrosamines	^216^
Ranitidine	LC-MS/MS	Bioanalytical method for quantification of N-nitroso­dimethylamine (NDMA) in human plasma and urine with different meals and following administration of ranitidine	NDMA	NA	15.625 pg mL^–1^	Novel and easily adaptable quantitation method	^217^
Valsartan & Losartan	LC-HRMS, GC-MS, LC-MS/MS	Performance characteristics of mass spectrometry-based analytical procedures for quantitation of nitrosamines in pharmaceuticals: Insights from an interlaboratory study	NDMA, NDEA, NMBA, NEIPA, NDIPA, NDBA	0.0008–0.04	0.0018–0.13	Capable of quantitating nitrosamines with acceptable accuracy, precision and detectability	^218^
Sartans	LC-APCI-MS/MS	Development, validation, and estimation of measurement uncertainty for the quantitative determination of nitrosamines in sartan drugs using liquid chromatography-atmospheric pressure chemical ionization-tandem mass spectrometry	NDMA, NMEA, NDEA, Npyr, Nmor, NDPA, Npip, NDBA	0.32–1.58 ng/mL	1.09–4.74 ng/mL	Rapid, selective, accurate, robust, and precise method for determining 8 nitrosamines	^219^
Valsartan	HPLC-MS/MS	Rapid analysis of genotoxic nitrosamines by HPLC-MS/MS	NDMA, NDEA, NDPA, NDBA, NPYR, NPIP, NMOR, NDELA	NA	0.1 ng/mL or 0.05 μg/g	Specific and selective quantitation method for 8 nitrosamine compounds	^220^
Ranitidine	LC-MS	Novel stability indicating LC-MS method for N-nitroso­dimethyl­amine genotoxic impurity quantification in ranitidine drug substance and drug product	NDMA	0.01	0.03	Precise, accurate and linear method. Can be employed for regular analysis	^221^
Rifampicin	LC-UHPLC-MS/MS	Comprehensive LC-UHPLC-MS/MS method for the monitoring of N-nitrosamines in lipophilic drugs: A case study with rifampicin	NDMA, MNP	NA	0.1 and 5.0 ng mL^–1^	Robust and highly suitable method for the quantification of N-nitrosamines in drugs	^222^
Metformin	LC-MS	A broadly accessible liquid chromatography method for quantification of six nitrosamine compounds and N,N-dimethylformamide in metformin drug products using high resolution mass spectrometry	NDMA, NMBA, NDEA, NDBA, NEIPA, NDIPA	NA	0.25, 1, 0.25, 0.25, 0.25, 0.25 ng/mL	Sensitive and selective method for quantitation of 6 nitrosamines	^223^
Telmisartan	LC-MS/MS	Ultrasensitive LC-MS/MS method for the trace level quantification of six potential genotoxic Nitrosamine impurities in telmisartan	NDMA, NDEA, NEIPA, NDIPA, NDBA, NMBA	9.6, 21.3, 37.2, 24.3, 58.7, 15.8	32.2, 65.6, 98.6, 65.7, 183.2, 49.1	Ultrasensitive and selective method for simultaneous determination of 6 nitrosamines. can be used for routine quantification	^224^
Sartans	LC-MS/MS	A multianalyte LC–MS/MS method for determination and quantification of six nitrosamine impurities in sartans like azilsartan, valsartan, telmisartan, olmesartan, losartan and irbesartan	NDMA, NDEA, NEIPA, NMBA, NDIPA, NDBA	NA	0.009	Sensitive and robust method for 6 nitrosamine impurities in 6 sartans. Can be used for routine quantification	^225^
Valsartan	HPLC, LC-MS/MS	Determination of N-nitroso­dimethyl amine impurity in valsartan by HPLC and LC-MS/MS methods	NDMA	0.0027, 0.0021 mg kg^–1^	0.0091, 0.0070 mg kg^–1^	Novel, rapid, sensitive and specific methods. LC-MS method is more sensitive and efficient than HPLC-DAD	^226^
Ranitidine	LC-MS	Determination of N-nitroso­dimethylamine in ranitidine dosage forms by ESI-LC-MS/MS; Applications for routine laboratory testing	NDMA	1.0 ng·mL^–1^	3.0 ng·mL^–1^	Valve switching technology, sensitivity and useful in quality control	^227^
Duloxetine hydro chloride	LC-MS/MS	Determination of potential nitrosamines NDMA, NDIPA and N-nitroso duloxetine in duloxetine hydrochloride by LC-MS/MS using APCI source	NDMA, NDIPA	0.0001	0.003	Simplicity, repeatability, sensitivity and suitable for the screening of samples in intended quality control applications	^228^
Valsartan & Irbesartan	LC-MS/MS	Development of a sensitive LC-APCI-MS/MS method for simultaneous determination of 11 nitrosamines in valsartan and irbesartan with a simple extraction approach	NDMA, NDEA, NMBA, DIPNA, NDBA, NMPhA, EIPNA, NMEA, NMIPA, NPIP, NPyR	0.001–0.008	0.008–0.05	Simple extraction, higher sensitivity and reproducibility	^229^
Olmesartan	HPLC-MS/MS	Development of an analytical method for the determination and quantification of N-nitroso­dimethylamine in olmesartan by HPLC-MS/MS	NDMA	0.04 μg/L	0.12 μg/L	Low LOD, LOQ, sensitivity and can be applied for other sartans	^230^
Rifampicin	LC-MS/MS	Trace level quantification of 4-methyl-1-nitrosopiperazin in rifampicin capsules by LC-MS/MS	4-Methyl-1-nitrosopiperazin (MNP)	0.0067	0.013	Sensitive, selective and effective method with good LOD and LOQ values	^231^
Metformin	LC-MS	In-use stability assessment of FDA approved metformin immediate release and extended-release products for N-nitrosodimethylamine and dissolution quality attributes	NDMA	1.72 ng/mL	5.23 ng/mL	Sensitive and selective method with very efficient extraction procedure	^232^
Sartans	LC-MS/MS	A multianalyte LC-MS/MS method for screening and quantification of nitrosamines in sartans	NDEA, NDELA, NDiPA, NDiPLA, NDMA, NDPA, NEIPA, NMBA, NMEA, NMOR, NPIP, NPYP	20.0 ng/g	50.0 ng/g	Screening and determination of 12 nitrosamines in sartan with sensitivity and selectivity	^233^
Sartans	LC-MS	Determination of genotoxic impurity N-nitroso-N-nethyl-4-aminobutyric acid in four sartan substances through using liquid chromatography–tandem mass spectrometry	NMBA	3.0 ng/mL	0.9 ng/mL	Fast, sensitive, stable, selective, reliable method for quality control use	^234^
Metformin	HPLC-MS	N-Nitrosodimethylamine formation in metformin hydrochloride sustained-release tablets: effects of metformin and hypromellose used in drug product formulation	NDMA	0.009	0.024	Sensitivity and selectivity	^235^
Valsartan	HPLC	Simultaneous estimation of six nitrosamine impurities in valsartan using liquid chromatographic method	NDMA, NMBA NDEA, NEIPA, NDIPA, NDBA	0.013, 0.011, 0.006, 0.011, 0.007, 0.011 μg/mL	0.041, 0.034, 0.020, 0.035, 0.022, 0.034 μg/mL	Simple, specific, precise, robust, and accurate method for quantitation of 6 nitrosamines	^236^
Rivaroxaban	LC-MS/MS	A critical N-Nitrosamine impurity of anticoagulant drug, rivaroxaban: synthesis, characterization, development of LC-MS/MS method for nanogram level quantification	N-(2-hydroxyethyl)-N-phenylnitrous amide	0.045 ng mL^–1^	0.15 ng mL^–1^	Sensitive, reliable, high-throughput method for routine analysis or quality control testing	^237^
Metformin	LC-HRMS, LC-MS	Rapid communication a cautionary tale: Quantitative LC-HRMS analytical procedures for the analysis of N-mitrosodimethylamine in metformin	NDMA	0.010 and 0.005 ng/mg	0.030 ng/mg and 0.010 μg/g	Comparison and evaluation performed among three methods (FDA1, FDA2 and private laboratory)	^238^
Valsartan	LC-MS	Development and validation of a single quadrupole LC/MS method for the trace analysis of six nitrosamine impurities in valsartan	NDMA, NDEA, NEIPA, NDIPA, NDBA, NMBA	NA	0.05	Capacity for detection and quantitation of 6 nitrosamines. Can be applied to commercial samples	^239^
Cigarette tobacco, cigar tobacco & smokeless tobacco	LC-MS/MS	Analysis of tobacco-specific nitrosamines in cigarette tobacco, cigar tobacco, and smokeless tobacco by isotope dilution LC-MS/MS	TNAs (NNN, NAT, NAB, NNK)	0.007 ng/mL	0.01 ng/mL	Simpler, faster and easily extended analysis of TSNAs in other samples	^240^
Tobacco & mainstream cigarette smoke	LC-MS/MS	Determination of tobacco-specific nitrosamines in tobacco and mainstream cigarette smoke using one-step cleanup coupled with liquid chromatography-tandem mass spectrometry	TNAs (NNN, NNK, NAT, NAB)	0.2–1.0 ng g^–1^, 0.1–0.3 ng cigarette^–1^	0.6–2.0 ng g^–1^, 0.2–0.6 ng cigarette^–1^	Simple and highly sensitive method. Solves the problem of matrix interference and tedious sample preparation faced by reference methods	^241^
E-Cigarette liquid and aerosol	LC-MS/MS	Analytical method for measurement of tobacco-specific nitrosamines in e-cigarette liquid and aerosol	TNAs (NNN, NNK, NAT, NAB)	4.40, 4.47, 3.71, 3.28 ng mL^–1^	NA	Identification and detection of 4 TNAs	^242^
Biopharmaceuticals	LC-MS/MS	A novel method for monitoring N-nitrosamines impurities using NH 2-MIL-101(Fe) mediated dispersive microsolid phase extraction coupled with LC-MS/MS in biopharmaceuticals	MeNP, NMOR, NPYR, NDEA, NPIP, NEIPA, NDPA, NDIPA, NMPA, NDBA, NDIBA, NDBzA	0.005–0.025 μg/L	0.010–0.250 μg/L	Avoids the sample pretreatment process for precipitating protein and concentrating by nitrogen sweeping, determination of 12 nitrosamines and can be applied for other aqueous matrixes (wastewater, and cosmetic products)	^243^
Ground water	LC-MS/MS	Simultaneous determination for nine kinds of N-nitrosamines compounds in groundwater by ultrahigh-performance liquid chromatography coupled with triple quadrupole mass spectrometry	NDMA, NMOR, NPYR, NMEA, NDEA, NPIP, NDPA, NDBA, NDphA	0.280–0.928 μg·L^–1^	MDL:^a^ 1.12–3.71 ng·L^–1^	Ability to effectively detect 9 types of N-nitrosamine compounds	^244^
Tailwater	SPE/SEC/LC-MS	An online-SPE/SEC/LCMS method for the detection of N-nitrosamine disinfection byproducts in wastewater plant tailwater	NDMA, NEMA, NPyr, NPip, NMor, NDEA, NDPA, NDBA, NDPhA,	0.12–6.60 ng/L	0.40–21.9 ng/L	Accurate quantitative and high compatibility method. Alleviates tedious human labor and can effectively overcome the matrix effect	^245^
Sartans, metformin & ranitidine	GC-MS	Determination of N-nitrosodimethylamine and N-nitrosomethylethylamine in drug substances and products of sartans, metformin and ranitidine by precipitation and solid phase extraction and gas chromatography–tandem mass spectrometry	NDMA, NMEA	0.3 and 0.07 μg/kg	0.9 and 0.3 μg/kg	First method for simultaneous determination of NDMA & NMEA in 8 drug substances and drug products. Can be used as a routine method	^246^
Valsartan	GC-MS/MS	Development of GC-MS/MS method for simultaneous estimation of four nitrosoamine genotoxic impurities in valsartan	NEIA, NDIPA, NDEA, NDMA	0.02–0.03	0.06–0.09	Satisfactory sensitivity, selectivity and suitability for quantification	^247^
Metformin	GC-HRAM-MS	Dispersant-first dispersive liquid–liquid microextraction (DF-DLLME), a novel sample preparation procedure for NDMA determination in metformin products	NDMA	NA	188 pg mL^–1^ & 52 pg mL^–1^	Sensitive, reliable, robust and novel sample preparation approach	^248^
Pharmaceuticals	GC-MS/MS	Evaluation and optimization of a HS-SPME-assisted GC-MS/MS method for monitoring nitrosamine impurities in diverse pharmaceuticals	NDMA, NDEA, NMEA, NEIPA, NDiPA, NDPA, NDiBA, NDBA, NPIP, NPYR, NMOR, NDiNA, NDCHA, NDPhA,	NA	0.05–0.25 ng/mL	Monitoring of 14 nitrosamine impurities in diverse pharmaceuticals	^249^
Ranitidine	GC-MS	HS-SPME-GC-MS as an alternative method for NDMA analysis in ranitidine products	NDMA	0.3 μg/L	1 μg/L	Proof of concept for using SPME as an eminent strategy to tackle the temperature problem in ranitidine analysis with low temperature, minimum preparation and extraction processes	^250^
Cilostazol, sunitinib and olmesartan	GC-MS	Development of a sensitive screening method for simultaneous determination of nine genotoxic nitrosamines in active pharmaceutical ingredients by GC-MS	NDMA, NMEA, NDEA, NDBA, NMOR, NPYR, NPIP, NDPA, *N*-methyl-npz	0.15–1.00 ng/mL	0.45–3.00 ng/mL	New, sensitive, simple, environmentally friendly method for extracting 9 nitrosamines from APIs. Can be used in routine quality control	^251^
Metformin	FE-SHSGC-NPD	A full evaporation static headspace gas chromatography method with nitrogen phosphorus detection for ultrasensitive analysis of semivolatile nitrosamines in pharmaceutical products	NDMA, NDEA, NEIPA, NDIPA, NDBA, NMPA, NMORP	0.1 ppb	0.25 ppb	Capability for high-throughput analysis and trace level nitrosamines analysis	^252^
Sartans	GC-MS/MS	Development of a sensitive and stable GC-MS/MS method for simultaneous determination of four N-nitrosamine genotoxic impurities in sartan substances	NDMA, NDEA, NDBA, NDIPA	0.002–0.150	0.008–0.500	Simple, suitable, sensitive, selective and satisfactory method for sensitive quantification of 4 nitrosamines	^253^
Ranitidine, metformin & nizatidine	GC-MS	Simultaneous determination of low molecular weight nitrosamines in pharmaceutical products by fast gas chromatography mass spectrometry	NDEA, NDMA, NDPh, NDPA, NMEA, NMOR, NPIP, NPYR, EIPNA, DIPNA, NMPA, MeNP	12 ng/mL	36 ng/mL	Precise, reproducible, linear, accurate and fast screening method for nitrosamines	^254^
Losartan	GC-MS	Development of a sensitive headspace gas chromatography–mass spectrometry method for the simultaneous determination of nitrosamines in losartan active pharmaceutical ingredients	NDMA, NDEA, DIPNA, EIPNA.	5, 5, 25, 25 ppb	25, 25, 50, 50 ppb	Efficient removal of potential interference, high-throughput routine analysis and can also be adapted for the simultaneous analysis of additional nitrosamines in other sartans	^255^
Ethalfluralin	SPE/GC-MS/MS	Determination of an overlooked deleterious source in pesticides	Ethyl-*N*-(2-methylallyl) *N*-nitroso amine (ΕΜΑΝΑ)	NA	0.33 μg g^–1^	Applied in routine analysis for postregistration control of plant protection products in the Greek market. The LOQ supersedes the limit set by EFSA (1 μg g–1) in the TAS	^142^
Water	GC-MS/MS	Determination of N-nitrosamines in water by gas chromatography coupled with electron impact ionization tandem mass spectrometry	Eight N-nitrosamines	0.76–2.09 ng/L	2.41–6.65 ng/L	Can be used to determine low (ng/L) levels of N-nitrosamines in water samples	^256^
Rat faeces	GC-MS	High level nitrosamines in rat faeces with colorectal cancer determined by a sensitive GC-MS method	NDMA, NMEA, NDEA, NDPA, NDBA, NPIP, NPIR, NDPHA	NA	0.5 ng/g	Sensitive and efficient method for detection of 8 nitrosamines in rat faeces	^257^
Children’s products	GC-MS	High resolution GC-orbitrap MS for nitrosamines analysis: Method performance, exploration of solid phase extraction regularity, and screening of children’s products	NDMA, NMEA, NDEA, NDiPA, NDPA, NMPhA, NDiBA, NEPhA, NPYR, NMOR, NPIP, NDBA, NDPhA, NDCHA, NDiNA, NDBzA	0.01–0.13 μg/kg	0.03–0.38 μg/kg	Highly accurate and sensitive method for detection of trace nitrosamines in complex matrixes	^258^
Sartans, metformin, ranitidine, sitagliptin pioglitazone, hydrochlorothiazide, amlodipine & vildagliptin	SFC-MS/MS	Analytical lifecycle management for comprehensive and universal nitrosamine analysis in various pharmaceutical formulations by supercritical fluid chromatography	NDMA, NDEA, NDELA, NEiPA, NDiPA, NDPA, NDBA, NMPhA, NMEPhA, NDPhA, NPyr, NPip, NMor, MNPaz, NMBA	NA	NA	Rapid, sensitive and versatile method for screening and investigation of nitrosamine impurities in various pharmaceuticals	^259^
Sartans	SFC	Simultaneous detection of nitrosamines and other sartan-related impurities in active pharmaceutical ingredients by supercritical fluid chromatography	NDMA, NDEA, NMEA, NDPA, NDBA, NDPhA, NPyr, NPip, NMor	4.55, 1.58, 1.81, 0.24, 0.34, 0.22, 3.71, 2.26, 4.20 ng/mL	NA	Highly sensitive method, can detect nitrosamines in the picogram to femtogram range and outperforms in terms of speed	^260^
Sartans	Micro SFC-MS/MS	Rapid quantitation of four nitrosamine impurities in angiotensin receptor blocker drug substances	NMBA, NDBA, NEIPA, NDIPA	NA	0.1 and 0.25	A high throughput automated method for screening and qualifying 4 nitrosamines	^261^
Rabbit serum	CE-ESI/MS	Determination of tobacco-specific N-nitrosamines in rabbit serum by capillary zone electrophoresis and capillary electrophoresis-electrospray ionization-mass spectrometry with solid-phase extraction	NNN, NAT, NAB, NNK, NNAL, iso-NNAL	0.1 and 0.2 mg/mL	NA	Better suited for the analysis of TSNAs in complicated biological samples for its sensitivity and extra information on molecular structure	^262^

a NA = Not available.

b Method detection limit.

## 10 Available Toxicology Data for N-Nitrosamine Impurities

N-Nitrosamine impurities, which are carcinogenic, mutagenic, and toxic, can potentially cause genetic mutations by metabolic interaction with deoxyribonucleic acid (DNA), even at very low concentrations. These impurities may also contribute to the development of cancer in humans. Therefore, it is crucial to assess, control, and eliminate N-nitrosamine impurities in all pharmaceutical medicines, taking into account their carcinogenicity, mutagenicity, and the threshold of toxicological concern, as outlined by the ICH M7 guidelines. The threshold of toxicological concern is deemed acceptable at 1.5 μg/day, according to these guidelines. Furthermore, this limit is considered negligible, posing no risk of cancer development in patients. In addition, the potential mutagenicity and carcinogenicity should be confirmed through an “in silico assessment” or predictions using two complementary (quantitative) structural activity relationship ((Q)­SAR) methodologies. These methodologies should be based on expert rule approaches and statistical methods.^263,264^

Moreover, it has been found that most of the N-nitrosamine impurities derived from PCCC (postcombustion CO_2_ capture technology) are toxic and carcinogenic to humans. In a similar vein, acute toxicity studies were conducted on fish and algae for N-nitrosamine impurities, with findings ranging between 3.2–5.85 mg/L. The most toxic effect on algae was reported by Bringmann and Kuhn, where the lowest observable effect concentration was 25 μg/L of N-nitroso-dimethylamine under chronic exposure. Furthermore, the genotoxicity and mutagenicity of many N-nitrosamine impurities have also been studied in both bacterial and mammalian cells.^265^

## 11 Current Status of Review on N-Nitrosamine Impurities in Various Drugs

Currently, numerous researchers have conducted comprehensive literature surveys and reported various review articles on N-nitrosamine impurities for method development, method validation, toxicology studies, allowable intake limits, regulatory guidelines, risk assessment, analytical methodologies, mechanism of formation of N-nitrosamine impurities, root of synthesis, sources of N-nitrosamine impurities, carcinogenicity, mutagenicity, etc. on several drugs. These drugs include all the sartan products (valsartan, irbesartan, candesartan, losartan, olmesartan, telmisartan, and azilsartan), ranitidine, and metformin products as well as substances found in water, food, beverages, cosmetic products, and tobacco. These products have been under scrutiny for quite some time due to numerous product recalls and the substantial impact on patients resulting from the presence of N-nitrosamine impurities, which are potentially toxic, carcinogenic, and mutagenic.^2,101,266–281^ Additionally, for other products like rifampicin, champix (varenicline), famotidine, nizatidine, and atorvastatin, it was observed that only a few researchers reported review articles on N-nitrosamine impurities.^9,101,263,264,266,271,281^ Unexpectedly, for several products, such as bumetanide, itraconazole, diovan, enalapril maleate, propranolol, lisinopril, duloxetine, rivaroxaban, pioglitazones, glifizones, cilostazol, and sunitinib malate, no review articles have been reported to date by any researcher. This indicates the necessity of reporting the presence of N-nitrosamine impurities in these products as well. Therefore, this review article will provide comprehensive information through various ways that includes the history, occurrence, acceptable intake limits, sources, mechanism of formation, pharmaceutical products containing N-nitrosamine impurities, product recalls, regulatory guidelines, available methodologies, toxicology data, mitigation strategies, and future recommendations regarding N-nitrosamine impurities in the above-mentioned products.

## 12 Available Software Programs to Predict or Confirm N-Nitrosamine Impurities

In the pharmaceutical and personal care industries, quantum mechanical and chemical approaches are generally used to predict the carcinogenic potency and DNA reactivity of N-nitroso impurities. This method is also known as computer-aided discovery and redesign (CADRE).^282,283^ Additionally, a web-based automated application has been developed to analyze the risk category of N-nitrosamine compounds from their SMILES notation, providing instant screening to identify high-risk formations of N-nitrosamine impurities.^284^ Furthermore, an in-silico risk assessment process is utilized to identify the risk of formation of N-nitrosamine impurities and other potential mutagenic impurities during the manufacturing of active pharmaceutical ingredients.^285–287^ The Food and Drug Administration has also published a new structural similarity method to identify surrogate compounds for assessing the carcinogenicity of N-nitrosamine impurities.^288^

## 13 Formation of N-Nitrosoamine Impurities from Excipients, Preservatives and the Formulation Process

The formation of N-nitrosamine impurities from excipients and preservatives and the formulation process is a significant concern in the pharmaceutical industry. Excipients are inactive substances used as vehicles while manufacturing the pharmaceutical products; however, these excipients can contain nitrosating agents like nitrites. These nitrites can react with secondary amines present in the drug to form N-nitrosamine impurities.^289^ In addition to the active pharmaceutical ingredients, there are several potential sources that can lead to contamination of the pharmaceutical products with N-nitrosamine impurities. These include impure excipients or solvents used during the drug product manufacturing process, the degradation of excipients, interactions between the drug and excipient that result in degradation, and the degradation of the active pharmaceutical ingredient during processing. Excipients are more complex in terms of structure and origins compared with the well-characterized active pharmaceutical ingredients. Excipients can originate from a wide range of sources, including animals, biotechnology, chemical synthesis, materials mining, and plant harvesting. The intricate nature and varied origins of excipients often lead to their impurities.^290^ Trace amounts of nitrosating impurities, such as nitrites and nitrates, are also found in commonly used excipients. These include pregelatinized starch, polyvinyl pyrrolidone, croscarmellose sodium, sodium starch glycolate, cross polyvinyl pyrrolidone, and lactose.^291,292^ Aldehyde is often found as an impurity in numerous pharmaceutical excipients. Further, aldehydes are proven to catalyze nitrosation with secondary amines. The secondary amines and their ammonium salts react readily with nitrite to form respective nitrosamines via iminium ion formation. In the case of PEG 300 and polysorbate 80, formaldehyde is produced as a result of the degradation of these polymers’ chains.^293^ Simultaneously, many cellulose excipients derived from plants contain furfur aldehyde due to the manufacturing process.^294^ Major excipient impurities, such as peroxides, hydrogen peroxide, formaldehyde, and formic acids, are proven to react with active pharmaceutical ingredients. Thus, it is important to understand the manufacturing pathway of excipients to identify potential components which can be associated with excipients; further, these can react with active pharmaceutical ingredients. Preservatives may also contribute to the formation of N-nitrosamine impurities if they contain or generate nitrosating species under certain conditions. The formulation process can also contribute to the formation of N-nitrosamine impurities, especially under acidic conditions or elevated temperatures; secondary amines can react with nitrosating agents to form N-nitrosamine impurities. The risk of N-nitrosamine impurity formation is particularly high when the drug substances are exposed, such as those containing secondary amines. Oxidation during the drying process is a high-risk event that can result in the formation of N-nitrosamine impurities. It has been demonstrated in the case of metformin tablets that the simultaneous presence of processing parameters (water and heat) and the nitrate and nitrite content of excipients play a crucial role in the formation of NMDA.^295^

## 14 Strategies for Mitigating the Contamination of N-Nitrosamine Impurities

Since N-nitrosamine impurities are potential human carcinogens, it is very important to strictly avoid the use of N-nitrosamine forming agents such as nitrating agents, secondary amines, and nitration catalysts during the synthesis and manufacturing of raw materials, starting materials, intermediates, active pharmaceutical ingredients, and drug products. This approach minimizes the risk of cancer development in humans. Regulatory agencies strive to provide clear instructions or mandates to drug manufacturers, advising against the use of these toxic materials during the manufacturing of active pharmaceutical ingredients. Furthermore, these agencies continuously monitor the synthesis routes of active pharmaceutical ingredients. If N-nitrosamine impurities are present in drugs and cannot be completely removed by various processes, then pharmaceutical manufacturers should implement a risk mitigation plan to ensure patient safety. Regulatory agencies can also regularly publish more guidelines and updates for pharmaceutical manufacturers (both active pharmaceutical ingredients and drug products) to minimize N-nitrosamine impurities contamination.

## 15 Future Recommendations

Considering the comprehensive literature survey, current research progress, regulatory guidelines, industrial status, and most importantly the patients’ needs concerning N-nitrosamine impurities, the following future recommendations can be proposed.

- Researchers can conduct further research on N-nitrosamine impurities, incorporating innovative and new ideas.
- Manufacturers of analytical instruments can develop more advanced, sensitive, and selective tools to enhance the detectability of N-nitrosamine impurities.
- Additional research can be conducted on analytical methodologies using sophisticated techniques for N-nitrosamine impurities.
- Pharmaceutical manufacturing companies should avoid using chemicals/reagents that produce nitrosamines during drug synthesis.
- Government organizations and institutions can focus their research on N-nitrosamine impurities.
- Regulatory authorities can pay more attention to N-nitrosamine impurities.
- Joint collaborations can be established among regulatory authorities, research institutions, and pharmaceutical companies to explore innovative ideas and approaches related to N-nitrosamine impurities.
- More advanced analytical methods can be developed for the detection and quantification of N-nitrosamine impurities from pharmaceutical products using cutting edge analytical techniques (LC-MS, GC-MS, CE-MS, etc.).

## 16 Conclusions

Considering their potential severity, N-nitrosamine impurities have become a widespread concern in the global regulatory landscape of pharmaceutical products. This concern arises due to their potential for contamination, toxicity, carcinogenicity, mutagenicity, and their presence in many active pharmaceutical ingredients, drug products, and other matrices. N-Nitrosamine impurities in humans can lead to severe chemical toxicity effects. These include carcinogenic effects, metabolic disruptions, reproductive harm, liver diseases, obesity, DNA damage, cell death, chromosomal alterations, birth defects, and pregnancy loss. They are particularly known to cause cancer (tumors) in various organs and tissues such as the liver, lungs, nasal cavity, esophagus, pancreas, stomach, urinary bladder, colon, kidneys, and central nervous system. Additionally, N-nitrosamine impurities may contribute to the development of Alzheimer’s, Parkinson’s, and type-2 diabetes in humans. Therefore, it is very important to control or avoid them by enhancing effective analytical methodologies using cutting-edge analytical techniques such as LC-MS, GC-MS, CE-MS, SFC, etc. Furthermore, these analytical methodologies should improve the sensitivity and selectivity, ensuring suitable precision and accuracy. This allows for the accurate detection and quantification of N-nitrosamine impurities in medications. Further, regulatory agencies such as the United States Food and Drug Administration (US FDA), the European Medicines Agency (EMA), the European Directorate for the Quality of Medicines (EDQM), the International Council for Humanization (ICH), the World Health Organization (WHO), the Agencia Nacional de Vigilancia Sanitaria (ANVISA-Brazil), etc. focused more on the hazards of N-nitrosamine impurities by providing appropriate guidance and updates regularly to drug makers and applicants. Similarly, drug manufacturers should avoid using nitrosating agents, secondary amines, and catalysts that have been proven to form N-nitrosamine impurities at various stages of the drug manufacturing process. These stages include the synthesis of raw materials, starting materials, intermediates, and final products as well as the manufacturing process for excipients. An avoidance strategy is more effective than a mitigation strategy in limiting or preventing the source of N-nitrosamine contamination in the final products.

This review comprehensively covers various aspects related to N-nitrosamine impurities. It includes information about their history, occurrence, acceptable intake limits, sources, formation mechanisms, pharmaceutical products containing N-nitrosamine impurities, product recalls, regulatory guidelines, available analytical methodologies, toxicology data, and mitigation strategies. Several review articles have been published recently by various researchers, focusing on N-nitrosamine impurities found in previously notified products including sartans, metformin, and ranitidine. These impurities have also been detected in a wide range of other products. Consequently, the primary focus of this article is on recently identified products that contain N-nitrosamine impurities such as rifampicin, champix (varenicline), famotidine, nizatidine, atorvastatin, bumetanide, itraconazole, diovan, enalapril maleate, propranolol, lisinopril, duloxetine, rivaroxaban, pioglitazones, glifizones, cilostazol, and sunitinib malate. As no review articles have been published on these products in the public domain, this article aims to fill that gap. Furthermore, the primary objective of this paper is to safeguard patients from cancer development by ensuring the use of safe and high-quality medicines. The detailed information provided in this work will be beneficial in achieving this goal.

## ⊥

Current institution address: 315 Riverbend Rd, Complex Carbohydrate Research Center (CCRC), University of Georgia, Athens, Georgia 30602, USA
